# Overestimation of ischemic core on baseline MRI in acute stroke

**DOI:** 10.1177/15910199231224500

**Published:** 2024-01-23

**Authors:** MA McArthur, E Tavakkol, M Bahr-Hosseini, R Jahan, GR Duckwiler, JL Saver, DS Liebeskind, K Nael

**Affiliations:** 1 Department of Radiological Sciences, 8783 University of California, Los Angeles, Los Angeles, USA; 2 Department of Neurology, 8783 University of California, Los Angeles, Los Angeles, USA

**Keywords:** Stroke, thrombectomy, magnetic resonance imaging

## Abstract

**Background and Purpose:**

In patients with acute ischemic stroke (AIS), overestimation of ischemic core on MRI-DWI has been described primarily in regions with milder reduced diffusion. We aimed to assess the possibility of ischemic core overestimation on pretreatment MRI despite using more restricted reduced diffusion (apparent diffusion coefficient (ADC) ≤620 × 10^−6^ mm^2/s^) in AIS patients with successful reperfusion.

**Materials and Methods:**

In this retrospective single institutional study, AIS patients who had pretreatment MRI underwent successful reperfusion and had follow-up MRI to determine the final infarct volume were reviewed. Pretreatment ischemic core and final infarction volumes were calculated. Ghost core was defined as overestimation of final infarct volume by baseline MRI of >10 mL. Baseline clinical, demographic, and treatment-related factors in this cohort were reviewed.

**Results:**

A total of 6/156 (3.8%) patients had overestimated ischemic core volume on baseline MRI, with mean overestimation of 65.6 mL. Three out of six patients had pretreatment ischemic core estimation of >70 mL, while the final infarct volume was <70 mL. All six patients had last known well-to-imaging <120 min, median (IQR): 65 (53–81) minutes.

**Conclusions:**

Overestimation of ischemic core, known as ghost core, is rare using severe ADC threshold (≤620 × 10^−6^ mm^2/s^), but it does occur in nearly 1 of every 25 patients, confined to hyperacute patients imaged within 120 min of symptom onset. Awareness of this phenomenon carries implications for treatment and trial enrollment.

## Introduction

Acute ischemic stroke (AIS) is the second leading cause of mortality and morbidity worldwide, with over 13 million new strokes occurring annually.^1^ Timely reperfusion is the most effective means to salvage brain tissue at risk of infarction and improve long-term outcomes, and endovascular therapy (EVT) has been added as an approved reperfusion treatment in patients with large vessel anterior circulation stroke since 2015.^2^

**Figure 1.**
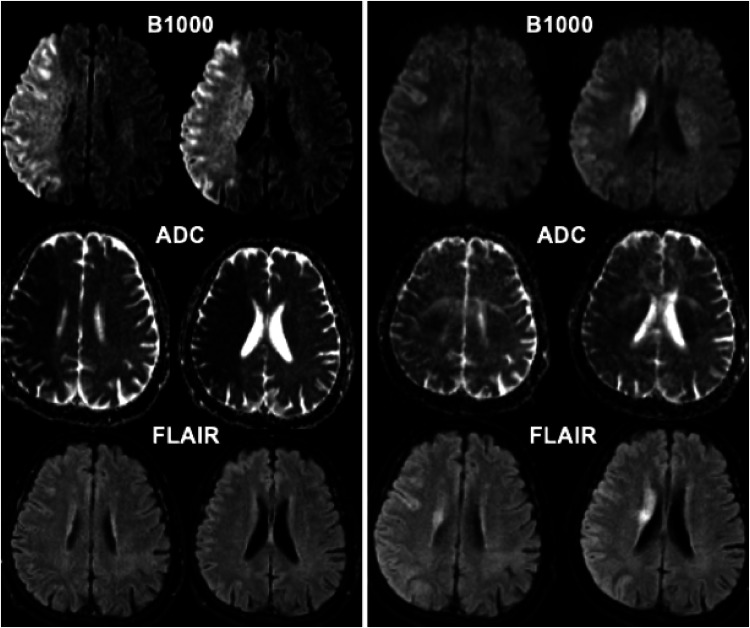
Baseline (left) and post-thrombectomy (right) MR images in a 60-year-old patient who presented with acute right MCA syndrome with M1 occlusion (not shown). Using ADC <620 volume of ischemic core on baseline imaging was calculated at 273 mL. MRI following successful reperfusion (mTICI 2b) obtained at 48 h post-thrombectomy shows significantly smaller infarct volume of 104 mL on B1000 images. ADC: apparent diffusion coefficient.

**Figure 2.**
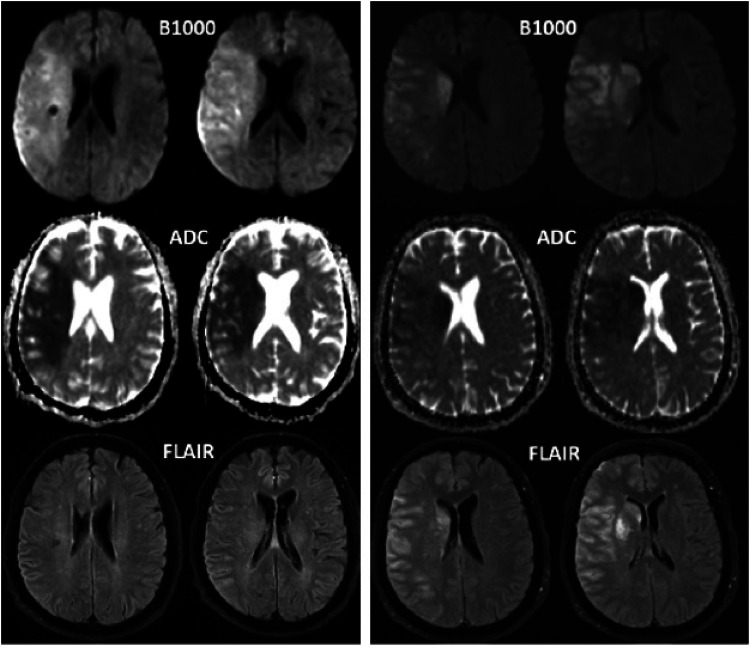
Baseline (left) and post-thrombectomy (right) MR images in a 69-year-old patient who initially presented with acute onset left-sided weakness and slurred speech. She was found to have right ICA occlusion with ischemic core volume calculated at 184 mL. The patient underwent successful reperfusion (mTICI 2b), and post-thrombectomy imaging revealed a significantly smaller infarct volume of 140 mL on B1000 images. ICA: internal carotid artery.

In the setting of clinical trials, it has been suggested that over-selection of patients may occur due to stringent selection criteria based on outcome predictions, resulting in underutilization of EVT in patients who may benefit.^3^ One such selection criterion, ischemic core volume, has been used to safely treat AIS patients with EVT. For example, the EXTEND-IA and DEFUSE 3 trials only enrolled patients with an ischemic core volume of <70 mL and the DAWN trial only included patients with ischemic core volume of <50 mL.^4–6^ The rationale for this limitation was that the treatment of irreversibly damaged tissue was not beneficial and may even result in cerebral edema or hemorrhage.^7^ More recently, restriction of ischemic core volume as an inclusion criteria for EVT has been challenged by the results of large core trials such as SELECT-2 and ANGEL-ASPECT, which show improved outcomes for patients treated with EVT versus medical management alone with ischemic core volume of ≥50–70 mL.^8,9^ However, accurate estimation of ischemic core on baseline CT or MRI continues to play a role not only for treatment decision but also for prognostic purposes, outcome prediction, and neuroprotective therapy development.

Overestimation of ischemic core on CT perfusion, also known as “ghost core,” has been reported in AIS particularly within a short time-window from stroke onset.^10^ In addition, overestimation of ischemic core from MRI using DWI has been described primarily in regions with mild reduced diffusion often defined by apparent diffusion coefficient (ADC) > 620 × 10^−6^ mm^2/s^ (between 621 and 760 × 10^−6^ mm^2/s^) rather than severe ADC ≤ 620 × 10^−6^ mm^2/s^ that is currently used in clinical practice.^11^ However, ghost core in patients with restricted ADC values of ≤ 620 × 10^−6^ mm^2/s^ has not been fully investigated. We aimed to assess the prevalence of ghost core on pretreatment MRI in regions with severe ADC hypointensity in AIS patients who were successfully treated with reperfusion.

## Methods

This retrospective study was conducted under an approved institutional review board. In this retrospective study, patients with AIS who presented to our comprehensive stroke center between 2015 and 2022 and met the following inclusion criteria were reviewed: (a) Had anterior circulation large vessel occlusion on pretreatment MRI; (b) Underwent successful reperfusion via endovascular thrombectomy (mTICI ≥2b); (c) Had follow-up MRI within 48 hours from treatment. This retrospective, single-center study complied with the Health Insurance Portability and Accountability Act and is approved by the institutional review board of this center.

MR imaging was performed on either a 1.5T MR scanner (Avanto, Siemens, Erlangen, Germany) or a 3T MR scanner (Trio, Siemens, Erlangen, Germany) within our hospital. DWI was acquired using a single-shot spin-echo EPI sequence using a TR range of 4000 to 5000 ms; TE range of 78 to 122 ms; matrix, 128  ×  128 mm; slices, 30  ×  5 mm. Diffusion gradients were applied along three orthogonal directions with b  =  0 and 1000 s/mm2. The FLAIR images were acquired using a TR range of 8000 to 10,000 ms; TE range of 88 to 134 ms; matrix, 256  ×  256 mm; slices, 30  ×  5 mm.

Baseline ischemic core volumes were derived from RAPID software (iSchemaView) using ADC ≤620 × 10^−6^ mm^2/s^ as part of routine clinical practice. Final infarct volume was calculated from follow-up MRI that was performed within 48 hours from the EVT time. Due to rise of ADC following reperfusion,^12^ final infarct volume was assessed by segmentation of DWI (B1000) images. Volume of infarction was calculated by a board certified neuroradiologist with more than 10 years of experience by applying a volume of interest on DWI, using a voxel-based signal intensity method subsuming the entire region of DWI hyperintensity.^13^

The ghost ischemic core volume was defined as an overestimation of ischemic core volume of more than 10 mL in comparison to final infarct volume. The baseline demographic, clinical, and treatment-related variables were further analyzed. For statistical analysis, Shapiro–Wilk test was used to determine normal distribution of data. Independent t-test or Mann–Whitney test were used as appropriate for comparative analysis of baseline clinical and demographic data between patients with and without ghost core (Figure 1).

## Results

Of the 156 patients reviewed, six patients (3.8%) had overestimation of ischemic core >10 mL. The baseline demographic, clinical, and imaging data for each patient with ghost core are summarized in Table 1. Comparison of common baseline clinical and demographic data between patients with ghost core (n  =  6) and without ghost core (n  =  150) are summarized in Table 2.

**Table 1.** Summary of demographic, clinical, and imaging data.

	1	2	3	4	5	6
Age (years)	76	43	69	35	60	68
Sex	F	F	F	F	M	F
NIHSS	18	23	16	0	19	20
Last known well-to-Imaging (minutes)	74	115	57	81	53	41
Time-to-reperfusion (minutes)	76	86	119	168	32	52
Etiology	Atrial fibrillation	Paradoxical Embolism	Unknown	Cardioembolism	Atrial fibrillation	Unknown
IV tPA	Yes	Yes	Yes	No	No	Yes
Type of thrombectomy	Clot retrieval	Clot retrieval	Aspiration, clot retrieval	Clot retrieval	Aspiration	Aspiration, clot retrieval
Clot location	Right M1	Left M2	Right ICA	Right M1	Right M1	Right M1
mTICI score	2B	2B	2B	2B	2B	2B
Baseline ischemic core (mL)	90.0	92.0	184.0	15.0	273.0	110.0
Final infarct volume (mL)	31.5	67.0	140.0	4.5	104.7	22.8
Overestimation volume (mL)	58.5	25.0	44.0	10.5	168.3	87.2

IV tPA: intravenous tissue plasminogen activator; ICA: internal carotid artery.

**Table 2.** Comparison of baseline clinical and demographic variables between patients with and without ghost core.

	Ghost core (n = 6)	Without ghost core (n = 150)	*p*-value *
Age (mean/SD)	58.5 (16.1) years	70.5 (13.8) years	0.09
Sex (M/F)	1/5	67/83	0.21
NIHSS (median, IQR)	18 (16–20)	15 (9–20)	0.48
Last known well-to-imaging (median, IQR)	65 (53–81) min	192 (66–528) min	0.06
Time-to-reperfusion (median, IQR)	81 (52–119) min	96 (72–140) min	0.24
Baseline ischemic core (Mean/SD)	127.3 (89.4) mL	18.9 (25.4) mL	0.001
Final infarct volume (mean/SD)	61.7 (52.4) mL	35.6 (40.4) mL	0.17

*Normal distribution was rejected for all variables, *p*-values were obtained by Mann–Whitney test

The etiology of the ischemic stroke was determined in 4/6 patients; two patients had atrial fibrillation without anticoagulation, one patient had paradoxical embolism, and one patient had cardioembolic stroke secondary to hypercoagulable state (cancer). Clot location was varied, with four patients having right M1 branch strokes, one with left M2 branch stroke, and one with right intracranial internal carotid artery stroke. Reperfusion therapy was intravenous tissue plasminogen activator plus endovascular thrombectomy (EVT) in four patients and EVT alone in two patients.

All six patients had last known well-to-imaging times less than 120 min, median (IQR): 65 (53–81) minutes. The time from last known well-to-reperfusion was median (IQR): 81 (52–119) minutes.

The average estimated ischemic core volume on baseline MRI was 127.3  ±  89.4 mL (mean  ±  SD) and final infarct volume on follow-up MRI was 61.7  ±  52.4 mL (mean  ±  SD). Of note, the estimated ischemic core volume on initial MRI of three patients was >70 mL, while the final infarct volume was <70 mL. The average overestimation of ischemic core volume was 65.6  ±  57.0 mL (mean  ±  SD).

The spatial distribution of ghost cores mainly involved the cerebral cortex and subcortical white matter in 5/6 patients. In one patient, the ghost core also involved the putamen. Interestingly, in all patients, some degree of deep gray matter involvement was present initially that persisted after treatment (not ghost).

## Discussion

In patients with AIS, ischemic core is often defined as brain tissue that is likely to be infarcted at the time of imaging regardless of treatment effect. Accurate assessment on non-invasive imaging remains challenging with no real gold standard.^14^ An estimated one million neurons inhabit just one voxel on DWI-MRI, and as such, current MRI techniques can only estimate the geographic region of neurons affected by infarction and not provide a true quantification of pan-necrosis.^14^ Nevertheless DWI-MRI remains the most sensitive and specific non-invasive imaging technique to evaluate the ischemic core by measuring cytotoxic edema in early minutes from the ischemic insult.^15,16^

While some degree of reversibility of DWI hyperintense signal following reperfusion therapies has been reported,^17^ ADC values at various thresholds have been tested and suggested that mild ADC reductions prior to complete metabolic energy failure may account for reversibility of reduced diffusion following reperfusion and subsequent neurologic improvement.^18^ Therefore, quantitative analysis of ischemic core has shown potential for overestimation using mild ADC values (ranging from 621 to 760 × 10^−6^ mm^2/s^). Purushotham et al. (2016) utilized a voxel-based analysis to demonstrate an ADC threshold of <620  ×  10^−6^ mm^2^/s provides a more accurate approximation of ischemic core without including potentially reversible penumbra.^19^ This supported earlier investigations,^20^ and hence an ADC threshold of <620  ×  10^−6^ mm^2^/s is currently used in routine clinical practice.

Our results demonstrate that ghost core, although rare by using low ADC threshold (<620  ×  10^−6^ mm^2^/s), does occur in approximately 3.8% of patients with AIS. This overestimation has potential clinical consequences; in our case series, 5/6 patients with ghost core had initial estimated volumes of >70 mL, while three of them had final infarct volumes of <70 mL. Although treatment eligibility based on the size of ischemic core is being challenged by recent large core trials such as SELECT-2 and ANGEL-ASPECT,^8,9^ this erroneous estimation of ischemic core may result in exclusion from treatment using current American Heart Association/American Stroke Association (AHA-ASA) guidelines.^21^ Interestingly, our patients who demonstrated ghost core vs. those without ghost core were on average 12 years younger (*p*  =  0.09), predominantly female (83%) (*p*  =  0.21), and had shorter time from last well known (*p*  =  0.06). These differences were trending towards, but did not reach, statistical significance most likely due to small and significantly imbalanced sample size (n  =  6 vs. 150).

Another important implication for quantifying ischemic core is to calculate infarct growth which has been a major target of neuroprotective trials. Following broad acceptance of EVT for AIS treatment, interest in neuroprotective trials has increasingly renewed.^22^ Currently several ongoing clinical trials are evaluating means of protecting brain from further damage in AIS patients ranging from medical therapeutics, remote ischemic conditioning, and direct electrical stimulation.^23,24^ Some of these trials have applied exclusion criteria regarding the size of ischemic core, and patients with large ghost core may thus be erroneously deemed ineligible for participation.^25,26^

Our study is limited due to retrospective design and relatively small sample size from one institution. Although MR acquisition parameters in our institution are comparable to what are broadly adopted by the stroke community, our image acquisition was performed using only two MRI scanners and therefore generalizability of our results should be interpreted in this context. To control for the effect of treatment on infarction size/growth, we had to include patients who had only successful reperfusion, and this further introduces selection bias in our results. While our study highlights the possibility of ghost core on MRI in early presenting AIS patients, a larger and prospective study is likely needed to further validate our results.

## Conclusion

Overestimation of ischemic core on MRI, known as ghost core, is rare using severe ADC threshold (≤620 × 10^−6^ mm^2/s^), but it does occur in nearly 1 of every 25 patients, confined to hyperacute patients imaged within 120 min of symptom onset. Awareness of this phenomenon carries implications for treatment and trial enrollment (Figure 2).
